# Inflammaging and Frailty Status Do Not Result in an Increased Extracellular Vesicle Concentration in Circulation

**DOI:** 10.3390/ijms17071168

**Published:** 2016-07-20

**Authors:** Ainhoa Alberro, Matías Sáenz-Cuesta, Maider Muñoz-Culla, Maider Mateo-Abad, Esperanza Gonzalez, Estefania Carrasco-Garcia, Marcos J. Araúzo-Bravo, Ander Matheu, Itziar Vergara, David Otaegui

**Affiliations:** 1 Multiple Sclerosis Unit, Biodonostia Health Research Institute—Donostia University Hospital, San Sebastian 20014, Spain; ainhoa.alberro@biodonostia.org (A.A.); matias.saenz@biodonostia.org (M.S.-C.); maider.munoz@biodonostia.org (M.M.-C.); 2 Spanish Network of Multiple Sclerosis, Barcelona 08028, Spain; 3 Primary Care Research Unit-Gipuzkoa, Osakidetza, Biodonostia Health Research Institute, San Sebastian 20014, Spain; maider.mateoabad@osakidetza.eus (M.M.-A.); ivergara@osakidetza.eus (I.V.); 4 Metabolomics Unit, CIC bioGUNE, CIBERehd, Technology Park of Bizkaia, Derio, Bizkaia 48980, Spain; egonzalez@cicbiogune.es; 5 Cellular Oncology Group, Biodonostia Health Research Institute, San Sebastian 20014, Spain; estefania.carrasco@biodonostia.org (E.C.-G.); ander.matheu@biodonostia.org (A.M.); 6 Computational Biology and Systems Biomedicine Group, Biodonostia Health Research Institute, San Sebastian 20014, Spain; marcos.arauzo@biodonostia.org; 7 IKERBASQUE, Basque Foundation for Science, Bilbao 48015, Spain; 8 Health Services Research on Chronic Patients Network (REDISSEC), Bilbao 48013, Spain

**Keywords:** extracellular vesicle, intercellular communication, human aging, inflammaging, frailty, interleukin-6

## Abstract

In the last decades extracellular vesicles (EVs) have emerged as key players for intercellular communication. In the case of inflammation, several studies have reported that EV levels are increased in circulation during inflammatory episodes. Based on this, we investigated whether aging results in elevated EV number, as a basal proinflammatory status termed “inflammaging” has been described in aged individuals. Moreover, we also hypothesized that frailty and dependence conditions of the elderly could affect EV concentration in plasma. Results showed that inflammaging, frailty or dependence status do not result in EV increase, at least in the total number of EVs in circulation. These results open a new perspective for investigating the role of EVs in human aging and in the inflammaging process.

## 1. Introduction

Extracellular vesicles (EVs) are membrane-coated particles of endosomal or plasma membrane origin that are secreted to the extracellular environment. They play an essential role for indirect intercellular communication as their membrane and cytosolic proteins, lipids and genetic material can be transferred between cells [1]. Moreover, almost all cell types release EVs and they can be isolated from plasma and other body fluids.

EVs are released both in physiological and pathological conditions and they are implicated in many cellular processes. In particular, EVs play a role in various stages of the immune response and they have been related to inflammatory, autoimmune and infectious disease pathology. EVs can carry and display antigenic material and are able to trigger antigen presentation and modulate immune responses [2]. It has also been reported that there are increased concentrations of EVs in plasma during inflammatory processes, such as in cancer or autoimmune diseases [3,4].

One of the hallmarks of human aging is the chronic low-grade inflammation, the so called inflammaging [5], a phenomenon that modulates intercellular communication. The age-associated immune dysfunction and accumulation of senescent cells promote inflammatory signals, such as elevated secretion of proinflammatory cytokines and activation of NF-κB transcription factor. Among inflammaging, the most widely studied feature is the circulating concentration of interleukin-6 (IL-6). The concentration of this interleukin is normally low (or non-detectable) in healthy adults, while elevated levels of IL-6 have been reported in the elderly, with increasing concentrations in the very old [6,7]. Moreover, elevated IL-6 has also been associated to mortality in the elderly [8].

Despite all this knowledge, there are many aspects of inflammaging that have not been elucidated, as the implication of EVs in the process. In the present study, and based on the previously mentioned increase of circulating EVs during inflammatory episodes, we proposed that this could also be observed in aged individuals as a result of inflammaging.

Furthermore, we also designed an approach to evaluate if the concentration of EVs in plasma could be related to the frailty status of old people—frailty status as defined by the Barthel Index [9] and the Tilburg Frailty Index [10]. These tests are applied to evaluate and measure the frailty and dependence status of the elderly, which could also be related to an increased chronic proinflammatory condition.

## 2. Results

### 2.1. Frailty Status Classification of Aged Individuals

For the present study, adults of different age ranges and elder people were enrolled. Participants were classified based on their age. Additionally, aged individuals (79–92 years) were asked to complete the Barthel and Tilburg Frailty Index questionnaires and were further classified as Robust, Frail or Non-autonomous, as shown in Table 1.

### 2.2. Interleukin-6 (IL-6) Concentration Is Increased in the Elderly

**Figure 1:**
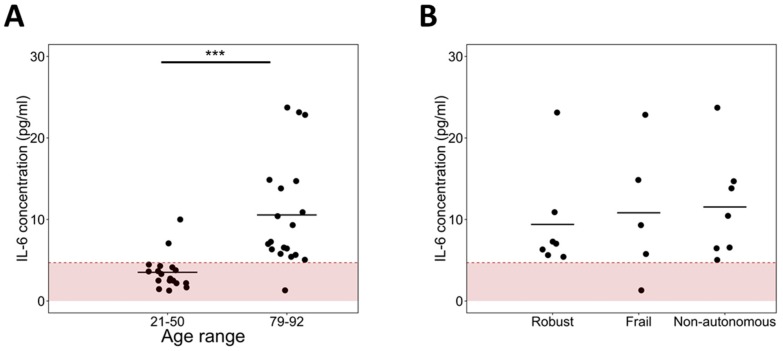
An elevated concentration of interleukin-6 (IL-6) is observed in aged subjects. IL-6 levels were measured by ELISA and concentration values above 4.7 pg/mL were considered detectable. (**A**) Elderly individuals have a higher concentration of IL-6 than adults (*** *p* < 0.001); and (**B**) there is a high variability among Robust, Frail and Non-autonomous elderly, but an increasing concentration with dependency can be observed. Each dot shows the concentration of a subject and the line represents the average value of the group.

The level of IL-6 in serum was measured and, obtained results demonstrated a very low, nearly non-detectable concentration in adults of different ages, while an elevated concentration in the elderly (*p* < 0.001) (Figure 1A). This result confirms the low-grade inflammatory condition of aged individuals. Additionally, when IL-6 levels of the elderly were compared depending on their frailty status, an increasing tendency with dependence was found (Figure 1B).

### 2.3. The Concentration of Extracellular Vesicles (EVs) Is Not Affected by Age and Frailty Status

**Figure 2:**
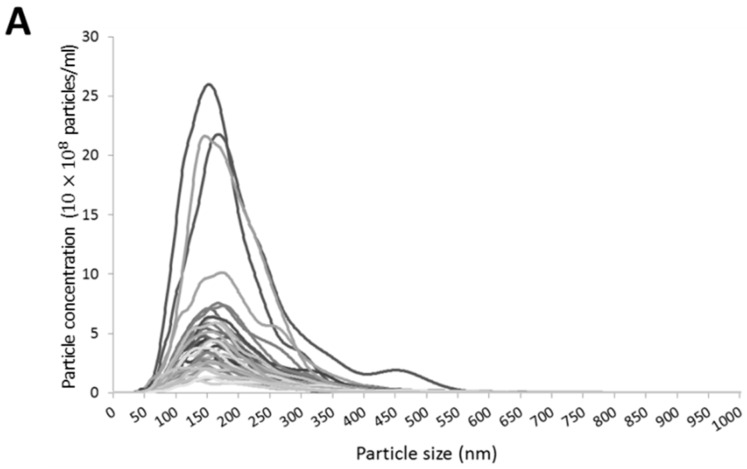

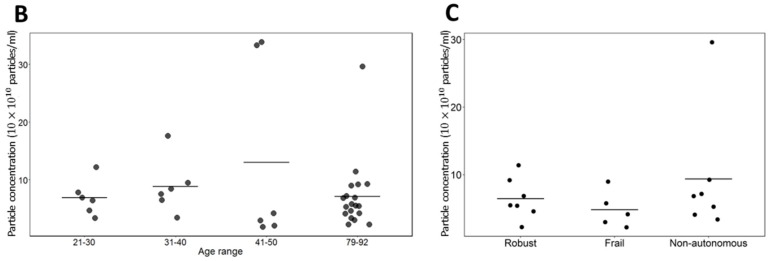
Nanoparticle tracking analysis (NTA) of extracellular vesicles (EVs). Particle size and EV concentration were measured by NTA. (**A**) Size distribution of EVs. Each line represents one sample. Despite the particle concentration difference, all samples have a similar size distribution—they are enriched in small EVs (50–300 nm); (**B**) EV concentration of different age ranges were compared and samples from elder people (79–92 years) do not show an increased EV number; and (**C**) among elder individuals, the frailty status does also not alter EV concentration. In figures (**B**) and (**C**) each dot shows the EV concentration of a subject and the line represents the average value of the group.

To assess the size profile and concentration of circulating EVs, nanoparticle tracking analysis (NTA) was conducted for all samples. Results showed that, regardless of particle concentration, all samples followed a similar EV size distribution, with most vesicles ranging between 50 and 300 nm in all instances (Figure 2A). This result demonstrated that our EV isolation protocol efficiently isolates small EVs, removing larger particles and platelets that can be found in plasma samples.

When comparing EV number, no significant differences were found between groups (*p* = 0.505), indicating that EV concentration is not increased with age (Figure 2B). Moreover, the concentration of EVs is also not affected by the frailty status of elder donors (*p* = 0.424), as shown in Figure 2C.

## 3. Discussion

During human aging, a chronic low-grade inflammatory state called inflammaging has been reported [6,7,8], and to our knowledge, this is the first report investigating, specifically, EV concentration in this process. The results presented in this work demonstrate that there are elevated IL-6 levels in the elderly, confirming the basal inflammaging. In contrast to what we hypothesized, and despite inflammaging, EV concentration in circulation is not affected by human aging. Moreover, frailty or dependence did also not alter the EV number. Many authors have previously studied the implication of EVs in diverse inflammatory processes, including cellular senescence, neurodegenerative diseases and cancer, indicating that both the total number of EVs in circulation and also EVs from specific cell origins can be increased [2,3,11]. Our results present a chronic inflammatory process—inflammaging—in which circulating EV levels are not affected.

In this work, and when studying EVs, there are several factors that should be taken into consideration. In our cohort, a high inter-individual EV concentration variability has been found within the same group. Similarly, previous experiments have demonstrated that the protein concentration and content of EVs differs depending on the donor [12]. On the other hand, specific medications may also affect EVs, as there are compounds that can modify EV production and release. For instance, immunomodulatory treatments can affect EV production by immune cells and modulate EV concentration in circulation [13,14,15]. This kind of effects should be considered when measuring EV levels especially in aged people, because nearly all of them have chronic medications. In this study, a representative sample of community-dwelling aged people was analyzed and, as expected, all were under chronic treatment. It was ethically not possible to ask to the participants in the study to interrupt their medications. Furthermore, the aim of our study was to evaluate whether the low-grade proinflammatory status was sufficient to alter EV concentration in the elderly, despite their medication. Moreover, even if the total number of EVs is not altered, EVs secreted from specific cell types could be affected, both in their concentration and cargo, modulating their function and effect in target cells, as described for other biological processes [11]. Finally, the limited number of samples in the study must be taken into account and results should be validated in a larger cohort.

In brief, these results represent a first report and demonstrate that there is no correlation between inflammaging and EV concentration in circulation. More extensive experiments are required to study the specific changes that occur to EVs in regard to human aging, and to further elucidate their role in the process.

## 4. Materials and Methods

### 4.1. Frailty Classification

A sample of community-dwelling aged individuals was classified by primary care services at the Donostia University Hospital, San Sebastian, Spain. Classification was made based on the multidimensional Barthel Index [9] and Tilburg Frailty Index [10], in their Spanish version. Physical, psychological, and social skills of each participant were assessed with these tests. First, the Barthel Index was applied to distinguish Autonomous (>90 points) and Non-autonomous elderly subjects. Then, Autonomous individuals were further characterized on Robust or Frail (>5 points) depending on their score in the Tilburg Frailty Index.

### 4.2. Blood Sampling and EV Isolation

Peripheral blood samples were collected at the Donostia University Hospital. All subjects gave written informed consent and the study was approved by the Hospital Ethics Committee. Donors underwent a questionnaire about disease/comorbidities and medication, fasting condition, exercising within the last hour, ovulatory cycle, acute illness, and last night sleeping hours. Donors with acute illness or recent exercising were excluded from the study. Comorbidities and medication of each individual were registered.

Samples were collected from 19 aged individuals (8 males and 11 females, mean age 83.73 years) and 18 adults—classified in three age ranges: 21–30, 31–40, and 41–50 years (three males and three females in each group). Samples were processed within the first hour and stored according to the criteria of the Donostia node of the Basque Biobank.

After discarding the first milliliter, blood collection was done by venipuncture with a 21-gage needle in a 4 mL EDTA tube and an 8 mL serum separator tube (Vacuntainer, BD Biosciences, Madrid, Spain). Serum separator tubes were allowed to clot for 30 min and centrifuged at 1400× *g* for 10 min to recover serum from the supernatant. EDTA tubes were kept upright and centrifuged at 1250× *g* for 20 min to recover plasma from the supernatant. EVs were isolated as described before by our group [16]. Briefly, plasma was centrifuged at 13,000× *g* for 2 min and supernatant centrifuged again at 20,000× *g* for 20 min to pellet EVs. The pellet was resuspended with 100 μL of filtered DPBS (GIBCO, Thermo Fisher Scientific, Madrid, Spain), filtered twice through a 0.22 μm-pore filter. Resuspended EVs and serum samples were stored at −80 °C.

### 4.3. Serum IL-6 ELISA Assay

IL-6 concentration was analysed by ELISA (BD Biosciences) following manufacturer’s intructions. Samples were measured in duplicate and results obtained with a microplate reader (Thermo Scientific Appliskan, Thermo Fisher Scientific, Madrid, Spain). IL-6 concentrations were calculated and values above the first standard (>4.7 pg/mL) were considered detectable.

### 4.4. Nanoparticle Tracking Analysis

The size distribution and concentration of EVs were measured using a NanoSight LM10 device (Malvern, Madrid, Spain) as described elsewhere [17]. Samples were diluted to appropriated levels to get accurate acquisitions (200–900 recorded tracks) [17] and camera settings were fixed and maintained for all samples. Filtered DPBS was tested and no background signal was detected. For each sample, two videos of 1 min were recorded and analyzed with NanoSight NTA software 2.2 (Malvern, Madrid, Spain). Data are shown as the average count of the two duplicates.

### 4.5. Statistical Analysis

Statistical analysis was performed with R version 3.2.2 (R Core Team (2015) [18]) in RStudio v0.99.486 (RStudio Team (2015) [19]). A Shapiro-Wilk test was applied to assess normality. As samples did not follow a normal distribution, Wilcoxon signed-rank test and non-parametric Kruskal–Wallis one-way analysis of variance were conducted to evaluate IL-6 and EV concentration differences between groups.

## 5. Conclusions

As previously described, IL-6 levels are increased in the elderly, demonstrating the basal low-grade inflammatory status of these individuals, a condition termed inflammaging. Conversely, the total number of EVs in circulation is not increased with age and frailty condition. Further investigations are needed to elucidate the implication of EVs in human aging and, specifically, in inflammaging.

## Figures and Tables

**Table 1:** Classification of enrolled individuals based on their age and frailty status. Samples of a total number of 18 adults and 19 elders were analyzed.

Group	Females	Males	Total
**Adults**			
21–30 years	3	3	6
31–40 years	3	3	6
41–50 years	3	3	6
**Elders (79–92 Years)**			
Robust	4	3	7
Frail	3	2	5
Non-autonomous	4	3	7
